# Vascular calcification burden of Chinese patients with chronic kidney disease: methodology of a cohort study

**DOI:** 10.1186/s12882-015-0132-3

**Published:** 2015-08-04

**Authors:** Zhi-Hong Liu, the China Dialysis Calcification Study (CDCS) Group

**Affiliations:** National Clinical Research Center of Kidney Diseases, Jinling Hospital, Nanjing University School of Medicine, 305 East Zhong Shan Road, Nanjing, 210016 China

**Keywords:** Chronic kidney disease, Mineral metabolism, Vascular calcification, End-stage renal disease, Dialysis

## Abstract

**Background:**

Vascular calcification is a common complication associated with chronic kidney disease and the major cause of cardiovascular disease in patients with end-stage renal disease. The vascular calcification risk burden is still unknown in China. This study aims to investigate the prevalence of vascular calcification and assess the predictive value of vascular calcification in patients with stage 5 chronic kidney disease on dialysis in China.

**Methods/Design:**

This is a national, multicenter, non-interventional, prospective cohort study planning to recruit 1520 patients with end-stage renal disease receiving hemodialysis or peritoneal dialysis for at least 6 months in 24 dialysis centers in China. All the patients provided written informed consents before participating in this study. It will include a baseline visit and 24 months follow-up period with 4 other visits at 6-month intervals. Vascular calcification images will be obtained to determine the prevalence of vascular calcification, coronary artery calcification, abdominal aortic calcification and cardiac valve calcification. Association between vascular calcification and all-cause and cardiovascular disease mortality and non-fatal cardiovascular events will be assessed. Disease management, as assessed by serum level of calcium, phosphorus and intact parathyroid hormone and its impact on vascular calcification, will also be surveyed.

**Discussion:**

The new results gained from this study will supplement limited current available data and provide better clinical decisions in Chinese patients with chronic kidney disease on dialysis.

## Background

Chronic kidney disease (CKD) is a critical health problem. It is estimated that there were nearly 120 million patients with CKD in China with an overall prevalence of 10.8 % [1]. It is similar to that in the United States (13.1 %) and Norway (10.2 %) but the awareness of CKD (10.04 %) is still relatively low in China [2–4]. The number of patients with end-stage renal disease (ESRD) is estimated to be 2 million in China, but less than 20 % of patients are undergoing dialysis [5].

Vascular calcification (VC) is a common complication associated with CKD and the major cause of cardiovascular disease (CVD) in patients with ESRD [6]. In contrast, patients without VC always have a better prognosis with limited progression of VC over a longer period of time [7]. The prevalence of VC increases as CKD progresses, from 40 % at stage 3 CKD to 80–99 % at stage 5 CKD on dialysis (CKD stage 5D) [8–12].

Mineral and bone disorder (MBD) is also a frequent complication of CKD associated with increased risk of VC, arterial dysfunction, morbidity and mortality [13]. A meta-analysis revealed that every 1.0 mg/dL of serum phosphorus was associated with 18 % increase of the risk of death in patients with CKD, indicating that hyperphosphatemia is an independent risk factor for mortality among those patients [14]. Hyperphosphatemia is increasingly considered the cause and a key factor inducing early and progressive VC in patients with CKD, implying that vigorous treatment of hyperphosphatemia with phosphate binders is essential to the prevention of VC [15, 16]. In addition, hyperphosphatemia is a key regulator involved in multiple mechanisms that induce and promote the progression of VC [15]. Therefore, prevention of MBD and lowering the circulating levels of phosphate and calcium have become major targets in the treatment of VC [17].

MBD management is not optimized for Chinese dialysis patients and hyperphosphatemia is an important issue needing greater awareness [18]. However, there is limited knowledge about the impact of VC on Chinese dialysis patients in current disease management and dialysis patterns. Additionally, the VC risk burden is still unknown in China, causing poorer prognosis and inadequate dialysis management. The China Dialysis Calcification Study (CDCS) is the first large, prospective, multicenter, observational cohort study to investigate the prevalence of VC using different non-invasive imaging techniques and to assess the predictive value of VC with associated risk factors for all-cause and CVD mortality in dialysis patients with CKD stage 5D in China.

## Methods/Design

### Study design

The CDCS is a national, multicenter, non-interventional, 24 month prospective cohort survey in CKD patients on dialysis. The study includes a baseline visit and 4 other visits at 6 month intervals. The estimated enrollment period is 10 months. It is planned to consecutively recruit 1520 patients with ESRD receiving stable hemodialysis (HD) or peritoneal dialysis (PD) for at least 6 months in 24 dialysis centers in China. Participating centers will be selected based on logistics constraints. Consecutive patients who fulfill the inclusion and exclusion criteria and are willing to participate will be included in the study (Table 1). Written informed consents were obtained from all patients before participation. Each site is expected to enroll approximately 30 patients. After that, enrollment will be competitive at all sites. This study will be able to mirror real life management of these patients. All statistical analyses will be performed using SAS 9.2 software (SAS Institute, Cary, NC, USA). All hypothesis tests will use two-sided tests with a significance level of 0.05.

**Table 1:** Inclusion and exclusion criteria of the CDCS

Inclusion criteria	Exclusion criteria
Male or female patients ≥18 years old	Patients’ life expectancy <6 months
Patients with ESRD receiving stable HD or PD for at least 6 months	Patients with acute kidney injury, active inflammatory diseases, parathyroidectomy or evident malignancies
Patient or legally accepted representative willing to sign Data Release Consent Form.	Patients with conditions making arterial calcification measurements technically impossible or unreliable, such as cardiac arrhythmias, amputations or severe peripheral vascular lesions
	Concomitant diseases that affect calcium status and soft tissue calcifications (sarcoidosis, multiple myeloma, HIV, amyloidosis)
	Pregnant or lactating women or women planning to become pregnant in the 6 months following entry into the study

*ESRD* End-stage renal disease, *HD* Hemodialysis, *PD* Peritoneal dialysis, *HIV* Human immunodeficiency virus

CKD is defined as abnormalities of kidney structure or function, present for >3 months, with implications for health, according to Kidney Disease: Improving Global Outcomes (KDIGO) 2012 Clinical Practice Guideline for the Evaluation and Management of CKD (Table 2) [19].

**Table 2:** Criteria for CKD (either of the following present for >3 months)

	Criteria
Markers of kidney damage (one or more)	Albuminuria (AER ≧30 mg/24 h; ACR ≧30 mg/g)
	Urine sediment abnormalities
	Electrolyte and other abnormalities due to tubular disorders
	Abnormalities detected by histology
	Structural abnormalities detected by imaging
	History of kidney transplantation
Decreased GFR	GFR <60 ml/min/1.73 m^2^ (GFR categories G3a–G5)

*GFR* Glomerular filtration rate, *AER* Albumin excretion rate, *ACR* Albumin-creatinine ratio

### Objectives

The primary objective for the CDCS is to determine the prevalence of VC in Chinese patients with CKD on HD or PD. The secondary objectives are:

1. To test the hypothesis that a greater severity of baseline VC is associated with a higher risk for all-cause and CVD mortality, and non-fatal CV events in Chinese patients with CKD on HD or PD.
2. To investigate the association between all-cause and CVD mortality, and non-fatal CV events and disease management (using serum levels of calcium, phosphorus and intact parathyroid hormone (iPTH), fibroblast growth factor 23 (FGF23) and related medications) in Chinese patients with CKD on HD or PD.
3. To investigate the impact of current disease management on VC.
4. To evaluate the control rate of patients achieving target goal of serum phosphorus, calcium and iPTH.
5. To validate the cardiovascular calcification index (CCI) in Chinese patients with CKD on HD or PD.

### Data collection

Visit 1 is at baseline/study entry and the follow-up includes 4 visits at 6 ± 1 month, 12 ± 1 month, 18 ± 1 months and 24 ± 1 month, respectively. The flow chart of detailed data and sample collection can be found in Table 3. At baseline, eligible patients will be evaluated for demographic information, smoking, alcohol drinking status, biochemical data, dialysis variables, concomitant medications, and personal and family disease history. Physical measurements including weight, height, body mass index (BMI) and vital signs (blood pressure and heart rate) will be obtained. Older age (male >55 years, female >65 years), arterial hypertension, family history (mother, father, brother, sister, children) of premature cardiovascular disease (≤55 years male, ≤65 years female), smoking, impaired glucose tolerance and/or impaired fasting glucose, dyslipidemia, and obesity are considered potent cardiovascular risk factors. The baseline biochemical data will be obtained using local routine laboratory methods performed within a week at the start of the study. If laboratory tests of serum 25-hydroxyvitamin D (25-OH Vit D) and FGF23 cannot be conducted in some centers, blood samples for testing 25-OH Vit D and FGF23 will be sent by the site study staff to those centers that can perform the assay. Blood sample collection will be performed according to the laboratory manual. Results from routine samples defined as samples taken within a week at the visit. Serum phosphorus, total calcium and iPTH will be tested at each visit. Target level of serum phosphorus, total calcium and iPTH in CKD stage 5 are defined according to Kidney Disease Outcomes Quality Initiative (K/DOQI) and KDIGO guidelines, respectively (Table 4) [20, 21]. Total serum calcium is adjusted if serum albumin is <4g/dL to better reflect the free calcium, using the formula proposed by the US Bone Metabolism Association: corrected total calcium (mg/dL) = total calcium (mg/dL) + 0.8 × [4.0 − serum albumin (g/dL)]. Date, time, primary reason and characteristics of death with autopsy results if applicable and any supportive documents that relate to fatal and nonfatal CV events are defined as clinical outcomes, and will be collected during the follow-up.

**Table 3:** Study flow chart of the CDCS

Procedures	Visit time (month)				
	Baseline	6 ± 1	12 ± 1	18 ± 1	24 ± 1
Data release consent form	●				
Demography data	●				
Inclusion/exclusion criteria	●				
Physical measurements (vital signs)	●				
Smoking and alcohol drink status	●				
Cardiovascular risk factors	●				
Disease history	●				
Concomitant medications	●	●	●	●	●
Hemoglobin, hematocrit	●				
Serum total calcium, phosphorus, iPTH	●	●	●	●	●
25-OH Vit D, FGF23	●		●		●
Glucose, HDL-C, LDL-C, TC and TG	●				
hs-CRP	●				
Serum albumin, ferritin, alkaline phosphatase	●				
Urea, uric acid, creatinine	●				
ABI	●				
Kt/V, dialysate calcium concentration	●				
Dialysis vintage, dialysis frequency and modality, membrane type	●				
EBCT or MDCT scan	●				●
Plain lateral lumbar radiograph	●				●
Echocardiography, LVMI/LVH	●				●
Clinical outcomes		●	●	●	●

*iPTH* Intact parathyroid hormone, *25-OH Vit D* serum 25-hydroxyvitamin D, *FGF23* Fibroblast growth factor 23, *HDL-C* High density lipoprotein-cholesterol, *LDL-C* Low density lipoprotein-cholesterol, *TC* Total Cholesterol, *TG* Triglyceride, *hs-CRP* High-sensitivity C-reactive protein, *ABI* Ankle-brachial index, *EBCT* Electron beam computed tomography, *MDCT* Multi-detector computed tomography, *LVMI* Left ventricular mass index, *LVH* Left ventricular hypertrophy

**Table 4:** Target level of serum phosphorus, calcium and iPTH in CKD stage 5

Biochemical parameters	K/DOQI	KDIGO
Serum phosphorus	3.5–5.5 mg/dL (1.13–1.78 mmol/L)	Toward Normal [2.5–4.5 mg/dL (0.81–1.45 mmol/L)]
Corrected total calcium	8.4–9.5 mg/dL (2.10–2.38 mmol/L)	Maintain Normal [Healthy: 8.5–10.5 mg/dL (2.13–2.63 mmol/L)]
Serum iPTH	150–300 pg/mL (16.5–33.0 pmmol/L)	2–9 times the upper normal limit (65 pg/mL, 6.9 pmmol/L)

*K/DOQI* Kidney Disease Outcomes Quality Initiative, *KDGIO* Kidney Disease: Improving Global Outcomes, *iPTH* Intact parathyroid hormone

### Imaging methods

VC images will be taken within a week of enrolment and the final visit of the study. Coronary artery calcification (CAC), abdominal aortic calcification (AAC) and cardiac valve calcification will be assessed by electron beam computed tomography (EBCT) or multi-detector computed tomography (MDCT), plain lateral lumbar radiograph and echocardiography, respectively. In addition, results of left ventricular mass index (LVMI) and left ventricular hypertrophy (LVH) through echocardiography will be taken within a week of enrolment and the final visit of the study as well.

CAC is measured by EBCT or MDCT as originally described by Agatston et al. [22]. Thirty to forty contiguous tomographic slices will be obtained at 3-mm intervals beginning 1 cm below the carina and progressing caudally to include the entire coronary tree. An attenuation threshold of 130 Hounsfield units (HU) and a minimum of 3 contiguous pixels will be used for identification of a calcific lesion. The density factor is assigned in the following manner: 1 for lesions with peak attenuation of 130–199 HU, 2 for lesions with peak attenuation of 200–299 HU, 3 for lesions with peak attenuation of 300–399 HU, and 4 for lesions with peak attenuation >400 HU [22]. Total CACS is determined by summing individual lesion scores from each of four anatomic sites (left main, left anterior descending, left circumflex, and right coronary artery). The CACS will be divided into approximate tertiles (0, <100, ≥100, ≥400 and ≥1000). CAC progression is evaluated as a continuous variable by calculating the difference in Agatston score between the two CT scans divided by the time between imaging. The 2-year progression of CAC is categorized as follows: absent (≤25th percentile), moderate (25th-75th percentiles), and accelerated (≥75th percentile).

AAC is assessed by semiquantitative scoring of a plain lateral lumbar radiograph using previously validated 24-point aortic calcification scale as originally described by Kauppila et al. [23]. Midpoint of the intervertebral space above and below the vertebrae will be used to assess calcific deposits in the aorta adjacent to each lumbar vertebra (L1-L4), and lesion grading is based on a 0–3 scale: 0, no aortic calcific deposits; 1, small scattered calcific deposits (<1/3 of the longitudinal wall of the aorta); 2, calcific deposits ≥1/3 and <2/3; 3, calcific deposits ≥2/3 [23]. The scores, obtained separately for the anterior and posterior wall, result in a range from 0 to 6 for each vertebral level and 0 to 24 for the total AAC score. The AAC will be divided into approximate tertiles (0, <5, and ≥5, ≥16).

Cardiac valve calcification is measured by a standard two-dimensional M-mode color Doppler echocardiography. Cardiac valve calcification will be considered present when bright echoes of more than 1-mm thickness are seen on 1 or more cusps of the aortic valve, mitral valve, or mitral annulus. Thereafter, patients are divided into 3 groups according to number of calcified valves: patients without valve calcification, patients with a single (aortic or mitral) valve calcification, and patients with calcifications in both valves.

LVMI is calculated with echocardiography and normalized to body height as an index in g/m^2^. LVH is diagnosed when LVMI is >125 g/m^2^ in males or >120 g/m^2^ in females.

### CCI

Points of CCI are assigned for patients age (60–69 and 70 years, 1 and 2 points, respectively), dialysis vintage 2 years (1 point), aortic and mitral valve calcification (3 and 1 points, respectively), and abdominal aorta X-ray scores of 1–6 and 7 (2 and 4 points, respectively). Race, sex and pulse pressure do not contribute to the CCI. Therefore, the CCI ranges from 0 to 11 points.

### Demographic and baseline data analysis

Categorical data will be presented in contingency tables along with frequencies and percentages. Continuous data will be summarized with frequency (n), mean, standard deviation, median, 1st quartile, 3rd quartile, minimum and maximum. Descriptive analyses will be presented by groups (with VC vs. without VC, with CAC vs. without VC, with AAC vs. without VC, with cardiac valve calcification vs. without VC). For categorical variables, group comparison will be based upon the results of the Chi-square test (for 2 × 2 tables) or the Cochran-Mantel-Haenszel test (for r × c tables, where r or c is greater than 2). Chi-square test will be replaced by Fisher’s exact test if the expected frequency in any of the cells of the contingency table is less than 5. For continuous variables, group comparison will be based upon the results of the *t*-test or the Wilcoxon rank-sum test, depending on the distribution.

### Primary analysis

Prevalence of VC at baseline and its corresponding 95 % confidence interval (CI) will be presented. Prevalence of CAC, AAC and cardiac valve calcification at baseline and their corresponding 95 % CIs will also be presented.

### Secondary analysis

The key secondary analysis will be a comparison of survival (based on all-cause mortality) between patients with and without VC based upon the log-rank test performed at a significance level of 0.05. Hazard ratios and 95 % CIs will be calculated by a Cox proportional hazard regression model. In addition, the multivariable Cox proportional hazard regression model will be performed, using the stepwise method (*p* <0.05 as the inclusion criteria and *p* <0.10 as the exclusion criteria), considering with or without VC as the independent variable and the baseline variables with *p* <0.25 in univariate analysis as covariates, and hazard ratios and 95 % CIs for each factor will be presented. The same analyses will also be done for survival (based on CVD mortality) and non-fatal CV event free survival. For each of the above endpoints, the separate comparisons of patients with CAC, with AAC, with cardiac valve calcification to patients without VC will be made at a significance level of 0.05.

Association between disease management (including serum level of calcium, phosphorus and iPTH, and medications), 25-OH Vit D, FGF23, and all-cause/CVD mortality, non-fatal CV events will be identified with Cox proportional hazard regression model, considering disease management as the independent variables. Associations will be tested using the Wald test and described through the hazard ratios and 95 % CIs.

Association between disease management, 25-OH Vit D, FGF23, ankle-brachial index (ABI), and LVMI/LVH and the occurrence of CAC, AAC and cardiac valve calcification at baseline will be identified with the logistic regression model. The univariate logistic regression model will be performed separately, considering disease management, ABI, and LVMI/LVH as independent variables. Associations will be tested using the Wald test and described through the odds ratios and 95 % CIs.

Association between disease management, 25-OH Vit D, FGF23, ABI, and LVMI/LVH and severities (scores) of CAC, AAC and cardiac valve calcification at baseline will be identified with the linear regression model, considering disease management, ABI, and LVMI/LVH as independent variables.

Association between CAC progression and disease management, 25-OH Vit D, FGF23, ABI, as well as LVMI/LVH will be evaluated by ANCOVA as continuous variables and evaluated by ordinal multinomial logistic regression as categorical variables.

Receiver operating characteristic curve (ROC curve) will be performed to assess the ability of the CCI to predict the presence of CAC (lesion score ≥100, ≥400 or ≥1000), with the area under the curve (AUC), positive and negative likelihood ratios calculated.

### Interim analysis

After the last patient is enrolled and all the baseline data including calcification status are collected, the data management committee will perform an interim analysis to evaluate all baseline data.

### Ethical considerations

This study will be conducted in accordance with the principles established by the 18th World Medical Assembly (Helsinki, 1964) and all subsequent amendments. The study was approved by institutional review boards of all participating centers and registered with the Chinese Clinical Trial Registry (ChiCTR-OCH-14004447 at http://www.chictr.org/en/). Written informed consent for participation in the study will be obtained from all participants. Ethical approvals are provided by all participating centers.

### Determination of sample size

From the latest published annual data, the mortality rate of Chinese CKD dialysis patients is approximately 10 %, so we assume the 2-year mortality rate for Chinese CKD dialysis patients is around 20 % [24]. Assuming the ratio between patients with and without VC is 7:3, a two-sided log rank test with an overall sample size of 1520 subjects (of which 1064 are in the VC group and 456 are in the non-VC group) achieves 80 % power at a 0.05 significance level to detect a difference of 0.07 between 0.79 and 0.86—the proportions surviving in VC groups and without VC group, respectively. This corresponds to a hazard ratio of 0.6398. The proportion of patients lost during follow-up is predicted to be 20 %. These results are based on the assumption that the hazard rates are proportional. Therefore, 1520 subjects are to be recruited.

## Discussion

VC is a major contributor to CVD, which is a significant cause of mortality in ESRD. However, the burden and management of VC in Chinese patients with CKD on dialysis are still unknown. Given that clinical trials are considered the best way to provide evidence and related knowledge for guidance, it is necessary to design a high quality national observational study that is capable of addressing these issues.

The CDCS is a uniquely-designed observational cohort study seeking to collect detailed clinical, biochemical and laboratory data to inform the prevalence and impact of management of VC in Chinese patients with CKD on dialysis, and test the hypothesis that baseline VC is a predictor for all-cause/CVD mortality and non-fatal CV events. This study aimed to recruit well-characterized patients in order to avoid incidence-prevalence bias, and the sample size will be large enough to achieve multiple study objectives with adequate variability [25].

This study may be limited by the nature of the design as an observational study, in comparison with randomized controlled trials which can commonly provide stronger evidence in the efficacy of therapies. Since the proportion of patients receiving HD or PD cannot be predefined, the strength of comparing the outcome between the two groups may be weakened. In addition, collection of some prognostic factors (e.g., brain natriuretic peptide and troponin) for CVD was not included in the study design as these tests will not be applicable for every enrolled patient in the real-world clinical practice setting.

In conclusion, as the first large-scale Chinese cohort study of patients with CKD on dialysis, the CDCS will utilize unique data for VC prevalence, demographic characteristics and disease management of the Chinese dialysis population to supplement the limitation of currently available data. The new results gained from the CDCS will provide increasing awareness of the importance of calcium balance and the risks of calcification, enable better clinical decisions and outcomes in assessing VC and management of serum phosphorus, calcium and iPTH in Chinese CKD patients on dialysis for nephrologists and health care providers, and be supportive for new clinical trials among Chinese CKD patients in the future. Further analysis of this cohort and comparison with other cohort studies in western countries might provide useful insight regarding race discrepancies on VC and related disease management in CKD patients.

### Trial status

This trial is still recruiting on the day of submission.

## Abbreviations

CKD: Chronic kidney disease
ESRD: End-stage renal disease
VC: Vascular calcification
CVD: Cardiovascular disease
CKD stage 5D: Stage 5 CKD on dialysis
MBD: Mineral and bone disorder
CDCS: China Dialysis Calcification Study
HD: Hemodialysis
PD: Peritoneal dialysis
KDGIO: Kidney Disease: Improving Global Outcomes
iPTH: Intact parathyroid hormone
FGF23: Fibroblast growth factor 23
CCI: Cardiovascular calcification index
BMI: Body mass index
25-OH Vit D: Serum 25-hydroxyvitamin D
K/DOQI: Kidney Disease Outcomes Quality Initiative
CAC: Coronary artery calcification
AAC: Abdominal aortic calcification
EBCT: Electron beam computed tomography
MDCT: Multi-detector computed tomography
LVMI: Left ventricular mass index
LVH: Left ventricular hypertrophy
HU: Hounsfield units
CIs: Confidence intervals
ABI: Ankle-brachial index
ROC curve: Receiver operating characteristic curve
AUC: Area under the curve
